# Finite element analysis of female pelvic organ prolapse mechanism: current landscape and future opportunities

**DOI:** 10.3389/fmed.2024.1342645

**Published:** 2024-01-23

**Authors:** Miyang Yang, Chujie Chen, Zhaochu Wang, Jiaye Long, Runyu Huang, Wan Qi, Rong Shi

**Affiliations:** ^1^The First Clinical Medical College, The Affiliated People’s Hospital of Fujian University of Traditional Chinese Medicine, Fuzhou, Fujian, China; ^2^Department of Anorectal, The Affiliated People’s Hospital of Fujian University of Traditional Chinese Medicine, Fuzhou, Fujian, China; ^3^Department of Interventional Radiology, Inner Mongolia Forestry General Hospital, The Second Clinical Medical School of Inner Mongolia University for The Nationalities, Yakeshi, China; ^4^Department of Radiology, The Affiliated People’s Hospital of Fujian University of Traditional Chinese Medicine, Fuzhou, Fujian, China

**Keywords:** finite element analysis, pelvic organ prolapse, gynecology, pelvic floor dysfunction, mechanism

## Abstract

The prevalence of pelvic organ prolapse (POP) has been steadily increasing over the years, rendering it a pressing global health concern that significantly impacts women’s physical and mental wellbeing as well as their overall quality of life. With the advancement of three-dimensional reconstruction and computer simulation techniques for pelvic floor structures, research on POP has progressively shifted toward a biomechanical focus. Finite element (FE) analysis is an established tool to analyze the biomechanics of complex systems. With the advancement of computer technology, an increasing number of researchers are now employing FE analysis to investigate the pathogenesis of POP in women. There is a considerable number of research on the female pelvic FE analysis and to date there has been less review of this technique. In this review article, we summarized the current research status of FE analysis in various types of POP diseases and provided a detailed explanation of the issues and future development in pelvic floor disorders. Currently, the application of FE analysis in POP is still in its exploratory stage and has inherent limitations. Through continuous development and optimization of various technologies, this technique can be employed with greater accuracy to depict the true functional state of the pelvic floor, thereby enhancing the supplementation of the POP mechanism from the perspective of computer biomechanics.

## 1 Introduction

The pelvic floor, consisting of complex and interconnected supportive tissues and muscles, is designed to counteract the effects of gravity and intra-abdominal pressure, while providing essential support for the pelvic organs (1, 2). When this support system is compromised, pelvic floor dysfunction (PFD) may occur, including pelvic organ prolapse (POP) (3). Currently, POP has become a grave public health concern. Approximately 30% of women endure varying degrees of POP during their lifetime, with approximately 12.6% requiring surgical intervention (4, 5). As the prevalence continues to escalate, the rate of surgical intervention after the age of 70 approximates 11% (6, 7). Currently, it is widely acknowledged that the primary etiology of POP is attributed to damage to the pelvic floor support structures resulting from vaginal delivery. Numerous previous studies have demonstrated a direct correlation between parametrial-vaginal support structure injury and POP development (8, 9). However, the pathogenesis underlying the occurrence of POP remains unclear. The risk and related factors for POP are primarily based on expert opinions, with limited support from epidemiological and clinical evidence (10, 11). Previously, the main methods for studying the mechanism of POP involved ultrasound, CT, and magnetic resonance imaging (MRI) to conduct anatomical research on morphological changes in pelvic floor tissues and organs (12). With the progressive advancement of biomechanics research in the female pelvic floor, the application of finite element (FE) analysis has introduced novel perspectives to biomechanical research of POP. Currently, the majority of research in this field is dedicated to finite element models, which are based on the MRI of pelvic floor individual tissues and organs, as well as the measurement of related parameters (13–15). The research on the overall mechanical balance principle, stress-induced injury mechanism, and organ interactions in the pelvic support system is still in its preliminary exploration stage (16). The current finite element models have been tailored to individual patients, posing significant challenges in interpreting research results that involve a wide range of populations (17).

This review retrospects the development and current research status of FE analysis, discussing various computational aspects related to model creation, material modeling, and boundary condition settings. This article provides a review of recent research on the mechanical properties of the female pelvic system and modeling techniques for POP, addressing key challenges and issues in analysis and modeling. The primary focus is to analyze relevant literature published within the past 3 years, aiming to enhance our understanding of the underlying mechanisms contributing to POP.

## 2 What is finite element analysis?

The technique of FE analysis originated from the field of engineering design and analysis. It is a simulation technique that utilizes the principles of continuum mechanics and elasticity to describe the mechanical changes, displacement variations, and other effects resulting from applied forces on the studied object (18). It is a method that determines approximate solutions to complex problems by decomposing them into a finite number of interconnected small units that are mathematically easier to handle (19). By conducting calculations and analyses on various components, including their geometric forms, architectural structures, compositions, and load conditions, the ultimate step is to integrate and analyze the calculation outcomes such as stress, strain, and displacement. Generally, the finer the unit division, the more accurate the calculation results (20). With the rapid advancement of medical imaging technology and computer software engineering, FE analysis has been granted shorter cycle times and enhanced simulation capabilities. This technology has transformed into a quantitative analysis method capable of observing intricate biomechanical systems that are unable to be directly visualized within the human body (21). Currently, FE analysis has gained extensive application in orthopedics, dentistry, and ophthalmology. It provides significant assistance to clinical research by studying mechanical mechanisms, simulating surgeries, comparing treatment effectiveness, predicting surgical risks, and designing implants (22).

## 3 Construction of finite element analysis on the female pelvic system and prolapse

The FE modeling of the female pelvic system involves various pelvic organs, including the urethra, bladder, vagina, rectum, pelvic floor muscles, and surrounding connective tissues. The construction of a FE model for the female pelvic support system poses significant challenges due to variations in material properties, mass, and density among different organs and soft tissues. The limitations of early female pelvic floor system FE model construction stem from the reliance on manual measurements and visual observations for estimating organ geometry, which often introduces significant errors into the results. To tackle this issue, the current model construction often relies on high-resolution MRI images and employs computer-aided or semi-automated contour drawing techniques along with algorithms to generate more precise images (23, 24). In the investigation of female pelvic system and prolapse, there may be notable variations in quantitative outcomes among different computational models due to the highly specific nature of patients. Therefore, constructing precise data representation and interpreting results play a significant role in comprehending the pathophysiological mechanisms of prolapse (25, 26).

Early FE modeling of the pelvic floor support system primarily focused on elucidating the biomechanics and functionality of the pelvic floor muscles. Given the pivotal role of the levator ani muscle, extensive investigations have been conducted by previous researchers to explore its geometric shape and mechanical properties, with a particular focus on variations observed in normal individuals, vaginal delivery, and prolapse (27–29). Hoyte et al. initially developed a FE model of the levator ani muscle, elucidating the structural disparities between normal and prolapsed females (30). Martins et al. developed a FE model of the levator ani muscle using shell elements with realistic thickness to comprehensively understand and predict the specific factors contributing to structural damage in this region (31). According to the Humphrey model, this study reveals that passive pressure on the levator ani muscle often leads to maximum stress at the puborectalis muscle. This finding effectively elucidates the mechanism underlying levator ani muscle damage and subsequent development of POP induced by vaginal delivery. In order to more accurately assess the strain distribution during the levator ani muscle’s strain process, Hoyte et al. and Lee et al. subsequently employed a varying thickness method to evaluate the functionality of the levator ani muscle (32). Lee et al. developed a FE model of the levator ani muscle based on dynamic MRI images captured during rest, maximum contraction, and maximum strain (33). This model was then integrated with measurements of overall stress in various states of the pelvic floor using a perineometer with vaginal probe. The observed changes in pelvic floor pressure exhibited a consistent pattern that aligned with clinical outcomes. Hsu Y et al. proposed a novel approach utilizing MRI and model construction to accurately quantify the transverse thickness of the levator ani muscle, thereby successfully elucidating the previously unexplored relationship between defective and intact muscle regions for the first time (34). Larson et al. developed a FE model to investigate the impact of unilateral levator ani muscle defect and its associated “architectural distortion” on POP (35). This study suggests that in cases of levator ani muscle deficiency and structural deformity, significant alterations in the ventral arch may occur, potentially affecting the direction of support for the anterior vaginal wall and increasing the risk of anterior vaginal wall prolapse.

Recently, there has been a shift in the focus of female pelvic floor system FE analysis toward the development of comprehensive models encompassing pelvic organs, muscles, and ligaments. Additionally, emphasis is placed on analyzing overall stress conditions to more realistically simulate the mechanical state of POP. Rubod et al. pioneered the utilization of MRI image overlay techniques to establish a comprehensive three-dimensional equivalent FE model, incorporating pelvic organs such as the bladder, vagina, and rectum, for evaluating the impact of bladder pressure and rectal load on vaginal function (36). Luo et al. conducted a focused investigation on the vaginal, uterine, and surrounding ligament structures, successfully quantifying the alterations in ligament length and relative angles induced by POP within a comprehensive model (37). Giraudet et al. have successfully developed a comprehensive anatomical FE model, incorporating the levator ani and pelvic diaphragm muscles, based on an MRI scan of a female cadaver for the first time (38). Gordon et al. developed an enhanced and customizable FE model to simulate POP, enabling rapid construction of tailored FE models incorporating specific structural geometric variations that accurately represent the morphological changes associated with POP (39). This novel simulation platform facilitates the development of a tailored FE model based on individual patient characteristics, enabling investigation into the pathogenic mechanisms underlying POP. This approach offers valuable insights for personalized treatment strategies.

Significant advancements have been made in FE analysis based on the POP over the past 3 years (Table 1). Silva et al. employed the FE model for the inaugural time to simulate the impact of diverse implants and various anchoring points (simple stich- a set of four nodes and continuous stitch-a line of nodes) techniques on reinforcing or replacing the apex ligaments [uterosacral ligament (USL) and cardinal ligament (CL)] during transvaginal reconstructive surgery under Valsalva maneuver: under different anchoring points one to mimic the USLs and other one to mimic the cardinal ligament (CL) after their total rupture (100% impairment) and with 90 and 50% of impairment (40). This study revealed that the implantation of USL and CL implants resulted in a reduction of upward and downward displacement of the vaginal wall, comparable to values observed in the healthy model. However, when the total rupture of the USL and CL occurred, the CL implant presented better behavior than USL implant. Simultaneously, this study revealed that in comparison to the health model and injuries with rupture rates of 50, 90%, and total, simple stitching resulted in a higher displacement ratio than continuous stitching. Subsequently, the research team utilized the same model to simulate sacrocolpopexy repair by substituting two commercial synthetic mesh implants with distinct mechanical properties for the damaged USL (41). This study indicated that the apical support function appears to be partially established when a mesh is used to replace/reinforce the USL. When utilizing a high material stiffness mesh for the correction of 50, 90, and 100% USL impairment, the displacement amplitude of the vaginal anterior wall is reduced which demonstrates that mesh with a higher material strength exhibit superior performance in the modification of USL. This two research suggest that the choice of different anchoring points and the material properties plays a crucial role in the successful repair of apical prolapse through surgical implants, offering valuable guidance to clinicians for selecting an appropriate approach in the treatment of POP.

**TABLE 1:** Summary table of clinical observations on finite element models of pelvic organ prolapse in the past 3 years.

Organ tissues only exist in the finite element model	Materials; IAP	Contributions	References
①②③④⑤⑥⑦⑧⑨⑪⑭⑮⑯	Yeoh and Ogden constitutive models; 4 kPa (Valsalva)	The simple suture leads to a higher vagina supero-inferior displacement than the continuous suture.	Silva et al. (40)
⑯⑰	Yeoh constitutive model; Not applicable.	The attachment point between the pubococcygeus and the skeleton were the places with the highest probability of postpartum lesions.	Xuan et al. (43)
①②③④⑤⑥⑦⑧⑨⑪⑭⑮⑯	Yeoh and Ogden constitutive models; 4 kPa (Valsalva)	The implantation of stiffer mesh lead to better outcomes than the implantation of the lower stiffness mesh.	Silva et al. (41)
①②③④⑤⑥⑨⑱㉕㉖	Linear elastic materials; 0.015 MPa	The most prominent displacement is observed in the anterior-posterior direction of the upper segment of the vagina.	Liu et al. (46)
①②③④⑥⑰⑱	No mention; 0.2 MPa (Valsalva)	Laparoscopic sacrocolpopexy without a supracervical hysterectomy was associated with a higher strain on pelvic organs.	Silva et al. (41)
③⑩⑪⑫⑬	The Yeoh model; 6, 10, and 17 kPa (5, 50, and 95% Valsalva)	USL and CL played an important role in maintaining the biomechanical stability of the uterus.	Chen et al. (15)
①②③④⑥⑰⑱	Elastic materials; 10 kPa (Valsalva)	Reconstructed finite element models using thin-sectional high-resolution anatomical images (Chinese Visible Human, CVH).	Xu et al. (45)
①②③④⑤⑥⑯	Yeoh and Ogden constitutive models; 826.46, 3679.08, 8224.61, 14,516.37 Pa (supine, standing, supine Valsalva, standing Valsalva)	A two-dimensional finite element model of pelvic organ prolapse was constructed for the first time.	Xue et al. (14)
①⑯⑰⑱⑲⑳㉑㉒㉓㉔	Martins constitutive model; Not applicable	Incorporated biomechanical analysis of the perineum.	

①-pelvic bone; ②-bladder; ③-uterus; ④-rectum; ⑤-urethra; ⑥-vagina; ⑦-anus; ⑧-pelvic fascia; ⑨-arcus tendineus fasciae pelvis (ATFP); ⑪-uterosacral ligament (USL); ⑭-cardinal ligament (CL); ⑮-round ligament (RL); ⑯-broad ligament (BL); ⑯-pubourethral ligament; ⑰-lateral ligaments of the rectum; ⑱-levator ani muscle (LA); ㉕-pelvic floor muscles (coccygeus, iliococcygeus, pubococcygeus, and puborectalis muscle); ㉖-perineum (perineal body, external anal sphincter, ischiocavernosus, bulbospongiosus, superficial transverse and deep transverse perineal muscles); ⑫-rectum caudate muscle; ⑬-joint longitudinal muscle; ⑲-joint longitudinal muscle; ⑳-uterosacral ligament; ㉑-sacrotuberous ligament; ㉒-sacrospinous ligament; ㉓-obturator internus; ㉔-abdominal cavity.

Marine et al. constructed a FE model for the first time to address the supra-cervical hysterectomy with laparoscopic sacrocolpopexy (LSC) (42). This research compares LSC with and without supra-cervical hysterectomy for POP to test the hypothesis that hysterectomy could reduce the stress and strain of mechanical fields on pelvic organs and on apical supporting ligaments. The finding demonstrates a significant association between the absence of supracervical hysterectomy following LSC and elevated strain in pelvic organs, characterized by an asymmetric distribution of stress and strain. These biomechanical results provide partial elucidation for the etiology of LSC recurrence and pain in specific cases of prolapse.

Xuan et al. developed a FE model to summarize the functional characteristics of pelvic floor muscles (PFM) during the second stage of vaginal delivery (43). By simulating the mechanical damage caused by varying fetal biparietal diameter sizes during childbirth, this study reveals that the point of connection between the pubococcygeus and skeleton experiences peak stress and strain in PFM at one-half of the delivery period, thereby providing a valuable biomechanical supplement to comprehend the mechanism underlying POP. Moura et al. have developed a more sophisticated FE model for PFM to investigate the structural damages resulting from vaginal delivery (44). This study presents an innovative consideration of the mechanical role played by the perineum during delivery, which has been previously overlooked due to its inability to be visualized on MRI scans. The research suggests that the maximum principal stress is predominantly localized in the area close to the perineal body, indicating its significance as the primary site for obstetric anal sphincter injuries occurrence.

One of the most anatomically complete pelvic models to date, contains 24 different structures, based on the thin-sectional high-precision anatomical images (CVH-5) (45). The innovation of this model lies in its more detailed division of the pelvic structure based on CVH-5, which provides a solid foundation for future FE analysis to achieve enhanced refinement and accuracy. Furthermore, this model holds potential for further exploration in future research endeavors.

Liu et al. created a FE model using women without POP to examine the correlation between high intra-abdominal pressure and compliance of the pelvic floor support system (46). This study suggests that the posterior vaginal wall exhibits greater stability than the anterior wall under high intra-abdominal pressure. The superior portion of the vagina experiences significantly higher displacement compared to its inferior part, particularly in the anterior-posterior direction. This research findings present a novel approach that holds great potential for clinical prevention and targeted surgical intervention of POP.

Chen et al. specifically developed a FE model to simulate the uterus and its associated ligaments in order to evaluate the mechanical alterations of these ligaments under varying positions and intra-abdominal pressure (47). This study developed one of the most comprehensive FE models to date, encompassing the uterus and its associated ligaments, thereby further substantiating the pivotal role played by uterosacral ligaments (USL) and cardinal ligaments (CL) in upholding uterine biomechanical stability.

Xuan et al. first developed a two-dimensional FE model to investigate the biomechanical conditions of both healthy pelvic floor system and one affected by POP at four utero-vaginal angles (normal, 45, 90, and 135° of posterior rotation) under combined impairments (healthy and 50% combined CLs, USLs and LA muscles) and four abdominal pressure conditions (14). The research findings indicate that the maximum descent of the cervix occurs when the uterus is tilted at an abnormal angle of 90°, potentially leading to uterine prolapse and posterior vaginal wall prolapse.

## 4 Challenges and issues in finite element analysis

### 4.1 Construction of a refined finite element analysis

Currently, the development of FE models for pelvic floor structures predominantly relies on MRI data, with accurate imaging data serving as the fundamental basis for model optimization (48). The image stacking technique of MRI facilitates real-time observation of the geometric shape and physiological structure of organs in subjects, thereby enhancing the accuracy of FE modeling. However, the FE model generated by MRI still exhibits a limitation of low resolution, impeding the clear differentiation of various ligaments in the pelvic floor, as well as fine structures such as different types of muscles and connective tissues (49). Simultaneously, there lacks a distinct demarcation between the puborectalis muscle, pubococcygeus muscle, and iliococcygeus muscle in terms of anatomical dissection. The researcher’s simplification of levator ani unified entity for study purposes, disregarded the heterogeneity and orientation of muscle fibers within each group. Additionally, assigning identical material properties to all groups inevitably introduces a discrepancy between experimental results and real-world conditions. Diffusion-weighted imaging (DWI) can be employed for non-invasive evaluation of muscle fiber orientation across the entire field of view, making it a valuable tool in pelvic floor muscle research with promising outcomes. However, meticulous management of artifact generation is imperative during clinical procedures (50). While previous research has established the quantitative information to the qualitative information of the levator ani muscle anatomy, practical FE models quality still poses several challenges, including discrepancies in anatomical understanding among FE model constructors and overlapping attachment points of muscle fibers (51). These difficulties frequently arise during actual FE modeling, highlighting the current lack of comprehensive and accurate literature on FE models that can fully characterize this structure. These challenges highlight the imperative for future research in the female pelvic floor system and prolapse FE analysis to address.

### 4.2 Finite element mesh partitioning

Choosing the right FE mesh is crucial for building accurate FE models of pelvic floor tissue organs and conducting subsequent analyses. The primary challenge encountered in FE meshing of the female pelvic system lies in converting numerous irregular surfaces into simplified geometric shapes suitable for FE analysis (52, 53). Due to the intricate structure of pelvic organ tissues, the generated mesh often violates shape or angle tolerance limits or exceeds the maximum allowable number of elements. Consequently, attempts at automatically meshing geometric shapes in FE software typically meet failure (54). The optimal solution currently available for this issue is to simplify the geometry or partition it into multiple components for separate meshing (55). The third challenge arises from the anatomical overlap of organs in MRI images, which presents certain difficulties for FE meshing in POP modeling. Accurate FE meshing plays a pivotal role in computing precise FE models, exerting a significant influence on the quantitative outcomes of model analysis. When modeling female pelvic organs, issues such as FE mesh overlapping and penetration commonly arise (56). The fourth challenge lies in setting the conditions for contact. The key challenge in establishing contact conditions lies in precisely locating the surfaces or mesh elements involved in contact, as well as defining the types and relationships of the contacts within FE software. Additionally, apart from geometric and contact non-linearities, the inclusion of material non-linearities was necessitated by the hyperelastic behavior exhibited by the soft tissues of the pelvic floor organs, thereby augmenting the complexity involved in constructing a finite element model for these tissues and organs.

### 4.3 Determination of biological material characteristics

The model that requires deformation must possess both the anatomical structure and physical behavioral characteristics of soft tissues, particularly for simulating their complex non-linear deformation behavior. The elastic properties of soft tissues are contingent upon the micro or macro structural organization, and their biomechanical characteristics can undergo alterations due to muscle or ligament injuries, modifications in collagen fibers, or hormonal fluctuations during menopause (57). The material properties utilized in FE analysis primarily stem from cadaver data, as well as measurement data derived from existing models or literature reports. Although there exist notable disparities in the maximum stress, stiffness, and other elastic characteristics between postmortem tissues acquired through uniaxial or biaxial stretching experiments on excised tissue samples and living tissues, current methodologies still encounter challenges in accurately representing the material properties of soft tissues (50). The absence of comprehensive data regarding the interaction between muscles and pelvic organs, fascia, and ligaments, including their connectivity and sliding dynamics, poses challenges in establishing model boundaries and loading conditions. Consequently, further extensive basic mechanical experimental research is necessary to generate more realistic and advantageous data.

Considering the limitations, inverse FE analysis can be employed as an optimization algorithm to enhance the precision and specificity of biomechanical characteristics estimation by constructing various material constant models and validating their results using dynamic MRI. However, numerous scholars have obtained disparate values for material constants through inverse FE analysis, potentially attributed to variations in pelvic floor muscle thickness and morphology as well as applied pressure (58–61). Currently, this technology still acquires supplementary hardware and software support. In the future, further clinical research is imperative to standardize the material parameters of diverse pelvic floor structures.

### 4.4 Material selection for finite element analysis

The pelvic floor structure in women primarily comprises the bladder, rectum, vagina, uterus, and their associated musculature and ligaments. The material characteristics of each structure, however, exhibit significant variations (62). Soft tissues are commonly characterized as non-linear hyperelastic materials, and their characteristics can be represented by constitutive models for hyperelastic such as Fung, Mooney-Rivlin, Yeoh, Neo-Hookean, Ogden, Humphrey (31). Although anisotropic mechanical behavior is observed in various pelvic tissues and organs, computational models for this specific material type are currently lacking in the majority of research on the female pelvic system (63). Martins conducted a comparative analysis of seven constitutive models for hyperelastic to assess their accuracy in representing soft tissue materials, revealing that all models, except for model Neo-Hookean, represented commendable consistency between theoretical and experimental data. Notably, models Ogden, Yeoh, and Martins exhibited the highest level of agreement among them (64). The Yeoh model, a reduced-order polynomial model derived from the Mooney-Rivlin model, accurately predicts the anisotropic, incompressible, and non-linear behavior of soft tissues. Presently, it stands as the most widely employed model for FE analysis of diverse pelvic diseases (47, 65, 66). The material in this model is defined as being composed of embedded fibers that tend to be dynamically activated over time under appropriate loads (67). The advantage of this model lies in its capacity to accurately depict the reverse S-shaped stress-strain curve and replicate the characteristic behavior of a substantial increase in material stiffness during later stages of strain (65). Currently, the pathophysiological mechanism of POP has been found to involve alterations in fiber distribution and volume fraction within muscles and ligaments, thereby leading to modifications in their mechanical characteristics. Consequently, employing an isotropic hyperelastic material property model is deemed inappropriate for this particular case (68, 69). Therefore, meticulous consideration of the distribution and volume fraction of fibers inherent in the process of prolapse is crucial when modeling the POP (70). In order to improve FE modeling for a better understanding of tissue characteristics related to POP, it is necessary to further develop new MRI-based technologies so as to enhance the capacity for discerning intricate anatomical structures across diverse MRI image modalities.

## 5 The future of finite element analysis in pelvic organ prolapse

The FE model represents the mechanical characteristics and displacements of a specific point at a given moment, but it has limitations in accurately representing biological entities. Future advancements lie in developing dynamic FE models that possess self-adjustment, compensation, and adaptability capabilities. Firstly, we should investigate more novel imaging techniques such as DTI to augment MRI imaging of the female pelvic system to generate more precise geometric models of pelvic organs (71). Secondly, the precision of FE mesh partitioning should be enhanced to accurately divide irregular geometric shapes, allowing for mesh penetration and overlapping in order to meet the real POP scenarios. Thirdly, it is imperative to conduct additional reverse FE analysis in order to establish standardized material parameters for diverse pelvic floor structures. Fourthly, when conducting FE modeling, it is necessary to consider the distribution and quantity of fibers in the tissue of pelvic in order to construct a model that better reflects reality. Finally, due to the fact that the pelvic floor structure is an intricately complex entity, encompassing multiple muscle groups, and ligaments with their interplay of tension and nerve traction tolerance. This not only necessitates that the personnel responsible for constructing the FE model possess a comprehensive understanding of pelvic floor anatomy and proficient MRI interpretation skills, but also calls for their ability to exhibit creativity. Therefore, future research should focus on continuously expanding foundational data, optimizing algorithms, and seeking more realistic clinical scenario data simulations through strong interdisciplinary collaboration.

## Author contributions

MY: Conceptualization, Investigation, Supervision, Writing – original draft. CC: Conceptualization, Investigation, Writing – review and editing. ZW: Conceptualization, Investigation, Writing – review and editing. JL: Conceptualization, Investigation, Writing – review and editing. RH: Investigation, Supervision, Writing – review and editing. WQ: Supervision, Writing – review and editing. RS: Funding acquisition, Supervision, Writing – review and editing.
